# Liquid Biopsy for Pancreatic Cancer Detection Using Infrared Spectroscopy

**DOI:** 10.3390/cancers14133048

**Published:** 2022-06-21

**Authors:** Alexandra Sala, James M. Cameron, Cerys A. Jenkins, Hugh Barr, Loren Christie, Justin J. A. Conn, Thomas R. Jeffry Evans, Dean A. Harris, David S. Palmer, Christopher Rinaldi, Ashton G. Theakstone, Matthew J. Baker

**Affiliations:** 1 Department of Pure and Applied Chemistry, University of Strathclyde, Thomas Graham Building, Glasgow G1 1XL, UK; alexandra.sala@dxcover.com (A.S.); loren.christie@dxcover.com (L.C.); david.palmer@dxcover.com (D.S.P.); 2 Dxcover Limited, Royal College Building, Glasgow G1 1XW, UK; james.cameron@dxcover.com (J.M.C.); justin.conn@dxcover.com (J.J.A.C.); 3 Swansea University Medical School, Swansea University, Swansea SA2 8PP, UK; cerys.jenkins@cansenseltd.com; 4 Gloucestershire Hospitals NHS Foundation Trust, Gloucester GL1 2EL, UK; hugh.barr@nhs.net; 5 Institute of Cancer Sciences, University of Glasgow, Glasgow G61 1BD, UK; j.evans@beatson.gla.ac.uk; 6 Singleton Hospital, Swansea Bay University Local Health Board, Swansea SA2 8QA, UK; dean.a.harris@wales.nhs.uk; 7 Department of Pure and Applied Chemistry, University of Strathclyde, The Technology and Innovation Centre, Glasgow G1 1RD, UK; christopher.rinaldi@aureumdx.com (C.R.); ashton.theakstone@strath.ac.uk (A.G.T.)

**Keywords:** infrared spectroscopy, ATR-FTIR, pancreatic cancer, serum, adenocarcinoma, PDAC

## Abstract

**Simple Summary:**

Pancreatic tumors are extremely difficult to detect in their early stages due to the lack of early symptomatic evidence and fast, simple, non-invasive, and accurate diagnostic tests. Computed tomography (CT) scans are the usual first step in investigating suspected pancreatic cancer, but may not detect early tumors—especially in asymptomatic patients. Equally, the carbohydrate antigen (CA) 19-9 blood test is not specific to pancreatic cancer, as CA 19-9 levels can be raised in symptomatic patients with other non-malignant diseases, or with other tumors located in the surrounding area. In this proof-of-concept study, infrared spectroscopy was used to discriminate cancers versus healthy controls, as well as cancers versus symptomatic controls. Classification algorithms successfully discriminated the classes with high performances, accounting for detection rates of up to 92%. Simple, minimally invasive, and accurate approaches can represent a powerful aid in the achievement of pancreatic cancer’s earlier detection to improve patients’ prognosis and quality of life.

**Abstract:**

Pancreatic cancer claims over 460,000 victims per year. The carbohydrate antigen (CA) 19-9 test is the blood test used for pancreatic cancer’s detection; however, its levels can be raised in symptomatic patients with other non-malignant diseases, or with other tumors in the surrounding area. Attenuated total reflection Fourier-transform infrared (ATR-FTIR) spectroscopy has demonstrated exceptional potential in cancer diagnostics, and its clinical implementation could represent a significant step towards early detection. This proof-of-concept study, investigating the use of ATR-FTIR spectroscopy on dried blood serum, focused on the discrimination of both cancer versus healthy control samples, and cancer versus symptomatic non-malignant control samples, as a novel liquid biopsy approach for pancreatic cancer diagnosis. Machine learning algorithms were applied, achieving results of up to 92% sensitivity and 88% specificity when discriminating between cancers (*n* = 100) and healthy controls (*n* = 100). An area under the curve (AUC) of 0.95 was obtained through receiver operating characteristic (ROC) analysis. Balanced sensitivity and specificity over 75%, with an AUC of 0.83, were achieved with cancers (*n* = 35) versus symptomatic controls (*n* = 35). Herein, we present these results as demonstration that our liquid biopsy approach could become a simple, minimally invasive, and reliable diagnostic test for pancreatic cancer detection.

## 1. Introduction

Pancreatic cancer is the seventh highest cause of cancer death worldwide, accounting for 5% of the total cancer deaths, with over 460,000 per year [1]. In the UK, pancreatic cancer caused more than 10,000 deaths in 2020, being the fifth highest cause of cancer death [1]. The 5-year and 10-year survival rates for pancreatic cancer have not exceeded 1% since 1970 [2]. Emergency presentation is still the most common route to diagnose pancreatic cancer; over 40% of patients visit their physician more than three times before diagnosis [3,4]. Two separate studies conducted in 2005 and 2016 reported that the average of life lost to pancreatic cancer amounts to 12 years [5,6]; this figure is an estimate of the potential years of life lost due to deaths considered premature when compared to the worldwide standard life expectancy, which highlights the urgent need for earlier detection.

One of the challenges with early detection of pancreatic cancer is that patients invariably have inoperable (i.e., incurable) disease when they present with symptoms, and there is no robust, accurate, and simple diagnostic method that can be applied for the screening of asymptomatic patients or high-risk groups. The radioimmunoassay to detect circulating levels of carbohydrate antigen (CA) 19-9 is the first diagnostic step; however, this serum test has shown a low ability to discern pancreatic cancer from inflammations, pancreas-related pathologies, or other cancers in patients, giving rather high numbers of false positives in symptomatic patients, with positive predictive values in the range of 0.5 to 0.9% [7]. In addition, it has been reported that CA 19-9 is not expressed in 10% of the Caucasian population with Lewis-negative blood group phenotype, making the test impossible to use for diagnostic purposes in these individuals [8]. Recent studies have investigated tumor biomarkers to develop diagnostic tools to increase the rates of early diagnosis for this aggressive type of cancer [9]. However, these preliminary studies have still not been incorporated into the clinical pathway, due to the numerous challenges in clinical translation—partially of an analytical and diagnostic nature (e.g., sample storage/preparation, disease discrimination, and diagnostic models’ accuracy), but also of an economic nature (e.g., funding for development, and international markets’ requirements) [10,11,12].

Vibrational spectroscopy has gained increasing interest in recent years for the clinical diagnosis/detection of diseases such as sepsis [13], malaria [14,15], and several types of cancers [11,16], employing minimally invasive techniques for detection thanks to the use of small portions of tissues, cells, and/or biofluids—such as saliva, blood plasma, and serum [11,17]. Both Raman and infrared (IR) spectroscopy have displayed their potential for use in the clinical field through a vast amount of research and proof-of-concept studies [10,11,12,16], although not many studies have had a significant number of patients (above 25 cases) in both the disease and control groups [16].

Attenuated total reflection Fourier-transform infrared (ATR-FTIR) spectroscopy has proven its potential for diagnosing cancer using biofluids and machine learning algorithms [11,17]. In particular, the detection of brain cancer has been moving past the proof-of-concept stage to the clinical assessment phase, with optimal results amounting to a sensitivity of 83.3% and a specificity of 87.0% in a cohort of 104 recruited patients, and a sensitivity of 81% and a specificity of 80% in a cohort of 385 patients, with a sensitivity of 91% for glioblastomas [18,19]. These outputs were obtained using a retrospective cohort of 724 patients, with values of up to 93.2% for sensitivity and 92.0% for specificity acquired for the training set [18,19,20,21,22,23]. Recently, an update was published by Cameron et al., which included 603 recruited patients and explored the possibility of tuning classification models for the best sensitivity or specificity; the sensitivity-tuned model achieved sensitivity of 96% at 45% specificity, whilst the specificity-tuned model reached a specificity of 90% at 47% sensitivity, for discriminating brain tumors from non-cancerous symptomatic patients [24]. These brain cancer studies have also been investigated from a health economic point of view, showing great potential for the British National Health Service (NHS) for future translation of the technique in the clinical setting [25,26].

The research presented in this article demonstrates the potential of ATR-FTIR spectroscopy for pancreatic cancer detection. With the aid of machine learning algorithms, we herein successfully analyze 235 human blood serum samples to discriminate patients with pancreatic cancer in advanced and early stages versus healthy controls, as well as patients with pancreatic cancer versus symptomatic controls affected by comorbidities and/or confounding diseases. To our knowledge, this is the largest and most promising proof-of-concept study to date for the clinical use of ATR-FTIR spectroscopy in the detection of early and advanced stages of pancreatic cancer.

## 2. Materials and Methods

### 2.1. Sample Cohort

Our patient database consisted of human blood serum samples; 63 samples from patients with pancreatic cancer and 35 control samples from symptomatic patients (i.e., people presenting symptoms of pancreatic cancer and recruited, but then diagnosed as non-cancerous patients) were prospectively collected from Singleton Hospital (Swansea, UK), the Gloucestershire Hospital Foundation Trust (Gloucester, UK), and the Beatson West of Scotland Cancer Centre (Glasgow, UK), following and respecting the ethical criteria established for the PANSPEC project (Integrated Research Application System, IRAS, ID No. 238735), from the University of Strathclyde Ethics Committee (UEC 17/81, November 2017), and the UK Health Research Authority (HRA) and Research Ethics Committee (REC 18/SW/0027, January 2018). The prospective recruitment was conducted according to the PANSPEC study protocol. Inclusion criteria were that the patient must be aged 18 years or over, have suspicion of pancreatic cancer with subsequent histological confirmation or alternative diagnosis confirmation, and be willing to provide written informed consent. Exclusion criteria were if the patient was under the age of 18 and/or unwilling/unable to consent to trial participation and/or belonging to a vulnerable group (e.g., refugees, homeless, sexually exploited or abused subjects, etc.). An additional 37 serum samples from cancer patients, along with 100 healthy control samples, were retrospectively obtained from the commercial source Tissue Solutions Ltd. (Glasgow, UK). Only samples from confirmed pancreatic cancer diagnosis and healthy donors were included in this retrospective proof-of-concept study; the diagnosis needed to be unique (no concurring tumors at the moment of pancreatic cancer diagnosis), and each serum sample needed to be collected at diagnosis, before any treatment. Further details of the patients are summarized in Tables S1–S4 in the Supplementary Materials. Three different cohorts were analyzed in this proof-of-concept study, all of which were age- and sex-matched where possible. Table 1 summarizes the characteristics of each cohort.

### 2.2. Sample Preparation

All serum samples were fully thawed at room temperature (18–23 °C) before spectral collection, and the sample sets were randomized prior to analysis. For every patient, a 3 μL serum aliquot was spotted—using a calibrated 1–10 μL pipette (Gilson, UK)—onto sample wells no. 1, 2, and 3 of a disposable Dxcover^®^ Sample Slide (Dxcover Ltd., Glasgow, UK), which utilizes multiple silicon internal reflection elements (SIREs) as non-fixed ATR crystals [18]. Each sample spot was spread across the full well using the pipette tip, to ensure homogeneous deposition [27]. Well no. 0 was left clean as the designated portion for background collections. The prepared slides were then left to dry in an incubator unit (Thermo Scientific™ Heratherm™; Thermo Fisher Scientific, Waltham, MA, USA) at 33–35 °C and humidity rate of 10–11% for 1 h to ensure optimal drying [27,28,29].

### 2.3. Spectral Collection

All of the spectra were collected in a range of 4000–450 cm^−1^ at a resolution of 4 cm^−1^, with 16 co-added scans, using a prototype of the Dxcover^®^ Platform (Dxcover Ltd., Glasgow, UK) consisting of PerkinElmer^®^ Spectrum Two™ FTIR spectrometers (PerkinElmer, Waltham, MA, USA) fitted with Dxcover^®^ Autosamplers (Slides’ indexing unit combined with ATR accessory; Dxcover Ltd., Glasgow, UK). A background spectrum was taken on well 0 and consequently subtracted by the PerkinElmer^®^ Spectrum IR™ acquisition software (PerkinElmer, Waltham, MA, USA) from each sample spectrum acquired. Three spectra were acquired for each biological replicate (i.e., wells no. 1, 2, and 3), and three technical replicates were performed, resulting in nine spectra for each sample.

### 2.4. Data Analysis

#### 2.4.1. Pre-Processing Method

Data were pre-processed using RStudio© software with the in-house-developed code PRFFECT (v3) [30]. Different spectral pre-processing options were explored to improve the classification algorithms’ performance by using a systematic search. The optimal spectral pre-processing consisted of (i) baseline correction through extended multiple signal correction (EMSC) [31]—with a human pooled serum spectrum as a reference—to scale each data point, followed by (ii) selection of the fingerprint region (1800–1000 cm^−1^) only, (iii) vector normalization (VN) to mean-center and scale the data, and (iv) application of a binning factor of 8 to improve the signal-to-noise ratio and reduce the dataset’s dimensionality [32].

#### 2.4.2. Classification Models and ROC Analysis

Random forest (RF) [33], partial least squares discriminant analysis (PLS-DA) [34], and support-vector machine (SVM) [35] with a linear kernel were employed as classification tools in order to discriminate between cancer and healthy/symptomatic control samples. Receiver operating characteristic (ROC) analysis [36,37] was also employed to examine the diagnostic ability at different probability/diagnostic thresholds. Even though the principles behind each of the machine learning algorithms slightly differ, they all function to create a trained model that can then predict the class outcomes of a test set [17,32]. Within each classification method, the data cohort was split into a 70% training set and 30% test set by patient ID, so that all 9 spectra from each patient are in the same dataset. Model hyperparameters were tuned using 5-fold cross-validation on the training dataset before the optimized model was retrained on all of the training data and used to predict the outcomes of the test set. Further details on the model hyperparameters can be found in the Supplementary Materials. The diagnostic prediction for each patient was taken as the consensus of the predictions of all 9 spectra. For Cohorts A and B (Table 1), null sampling was chosen, as both classes were balanced. However, the synthetic minority oversampling technique (SMOTE) [38] was used as a sampling method to minimize bias in the classification’s results for the unbalanced dataset (Cohort C); this minimizes bias by artificially mixing the data in order to create “new” samples, aiming to achieve balance in the dataset [22]. All of the classifications and ROC analysis were also performed with PRFFECT (v3) [30]. To reduce sampling bias and to improve the estimate of the model’s performance, the classification statistics are reported as means over 51 different training and test set splits [22].

#### 2.4.3. Permutation Test

A total of 1000 permutations/bootstraps (i.e., statistical processes that consist of resampling a dataset to create new simulated samples) were also performed on each cohort to ensure the statistical significance of the discrimination between cancer and control samples, and they were carried out using “cluster-toolbox-v2.0”—a chemometric toolbox for MATLAB^®^ (The MathWorks, Natick, MA, USA) that is freely available online [39,40]. Each bootstrapping validation was based on PLS-DA classification with no subsampling (for Cohorts A and B, balanced), or on SMOTE [38] (for Cohort C). The statistical process allowed the training of a model with known labels (observed model) and of another with random permuted labels (null model); the accuracies of those models’ predictions were then divided into two different distributions with an associated *p*-value output assessing the statistical significance of the models’ performance [39].

### 2.5. Study Outcomes

This retrospective proof-of-concept study evaluates the ability of an ATR-FTIR spectroscopic liquid biopsy to discriminate between pancreatic cancer and healthy control patients, and between pancreatic cancer and symptomatic control patients. The spectral data were pre-processed and subsequently analyzed using classification algorithms and ROC analysis to extract measures of sensitivity, specificity, accuracy, and AUC; the classification outputs were validated through permutation tests. The study’s outcomes show that this technique can differentiate pancreatic cancer from both healthy and symptomatic control patients, suggesting a great potential for clinical implementation.

## 3. Results

### 3.1. Pancreatic Cancer Vs. Healthy Control Samples (Cohort A)

**Figure 1:**
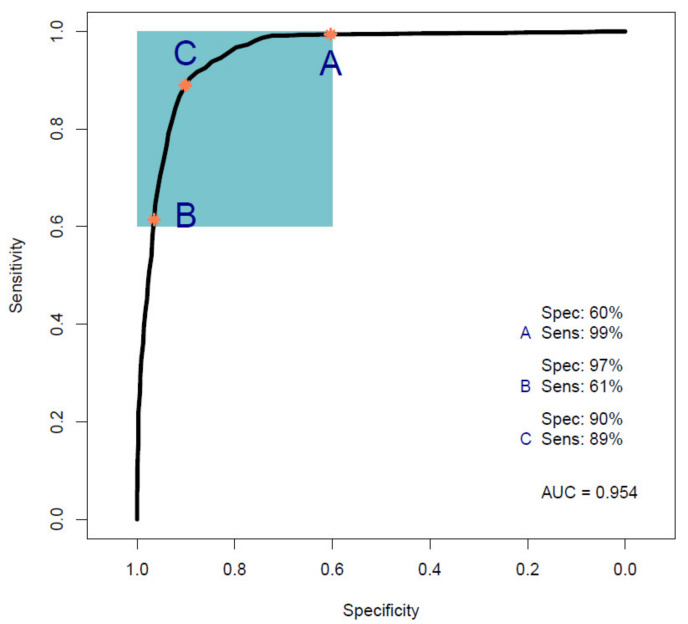
Receiver operating characteristic (ROC) curve analysis calculated from a partial least squares discriminant analysis (PLS-DA) model performed on Cohort A. The colored box indicates the region with sensitivity and specificity greater than 60%. The orange dots indicate the points within that region that have maximum sensitivity (A), maximum specificity (B), and balanced sensitivity and specificity (C) (Sens, sensitivity; Spec, specificity; AUC, area under the ROC curve).

**Figure 2:**
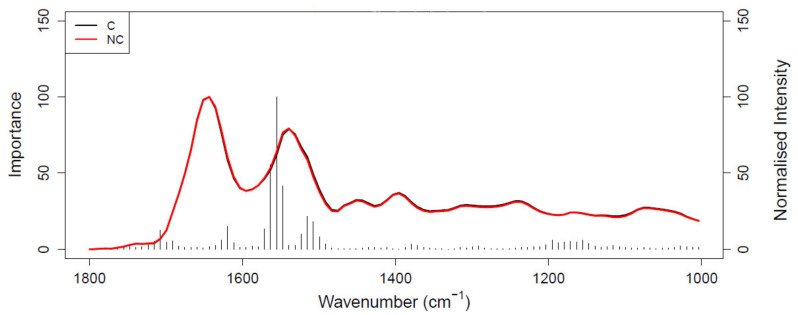
Gini importance plot of random forest (RF) analysis performed on Cohort A; C (black) represents the pancreatic cancer spectrum and NC (red) represents the healthy control spectrum.

**Figure 3:**
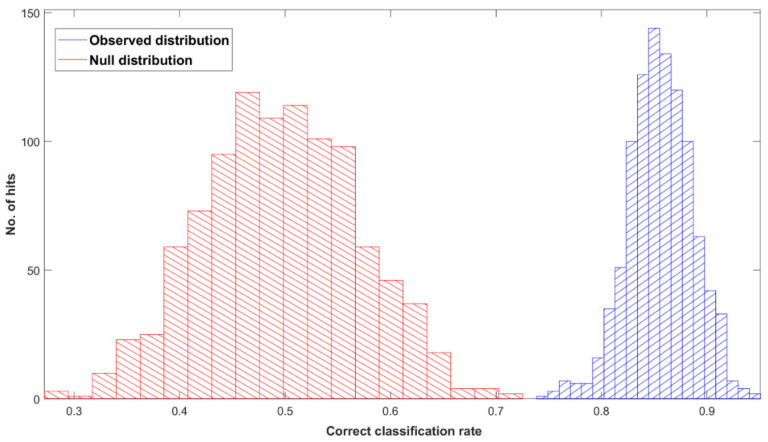
Permutation tests’ output plot; null (red) and observed (blue) distribution classification rated for Cohort A with a partial least squares discriminant analysis (PLS-DA) classification model after 1000 bootstraps, using pre-processed spectra.

Figure 1 shows the ROC curve calculated from the best-performing model (i.e., PLS-DA; Table 2) discriminating between 100 pancreatic cancer and 100 healthy control samples. With a balanced sensitivity and specificity point of 90% and 89%, respectively, and an area under the curve (AUC) equal to 0.954, it proves an excellent degree of diagnostic separability [37]. The curve shows a high grade of symmetry with a good balance between sensitivity and specificity, properly pertaining to the great accuracy of the model [37]. The analysis also explored the possibility of higher sensitivity (99%) and specificity (97%) when altering the diagnostic thresholds to a minimum specificity and sensitivity of 60%. The results showed a solid and clear distinction between cancer and control samples, confirmed also by RF, PLS-DA, and SVM performed on Cohort A, resulting in overall sensitivity ranging between 84.4 ± 7.2% and 91.0 ± 5.4%, and specificity between 86.3 ± 7.1% and 87.8 ± 6.2%, with an accuracy between 85.4 ± 4.5% and 89.7 ± 2.9% (Table 2). The best-performing algorithm was PLS-DA, with accuracy of 89.7 ± 2.9%, while RF seemed to have a lower performance rate, although still scoring above 84% in all of the statistical metrics considered. Both the PLS-DA and SVM classification methods followed an equal trend of slightly higher sensitivity than specificity, while RF showed a slightly higher specificity. The RF Gini importance plot in Figure 2 [33,41] confirms the ROC analysis and classification results, highlighting the most important discriminatory features between ~1570 and ~1500 cm^−1^, on both sides of the amide II peak (δ(NH)/ν(CN)). A table containing the top 15 wavenumbers in order of importance, with corresponding tentative spectral assignment, is reported in the Supplementary Materials (Table S5) [22,42,43,44]. The clear discrimination is also confirmed by the permutation test plot in Figure 3, which shows no presence of overlap between the null distribution and the observed distribution, proving the separation to be statistically significant, with an optimal *p*-value < 0.0001 [39]. The amide II region has been already documented as one of the key spectral portions for cancer detection (e.g., brain tumors) [22,33,44].

### 3.2. Pancreatic Cancer Vs. Symptomatic Control Samples (Cohorts B and C)

#### 3.2.1. Cohort B

**Figure 4:**
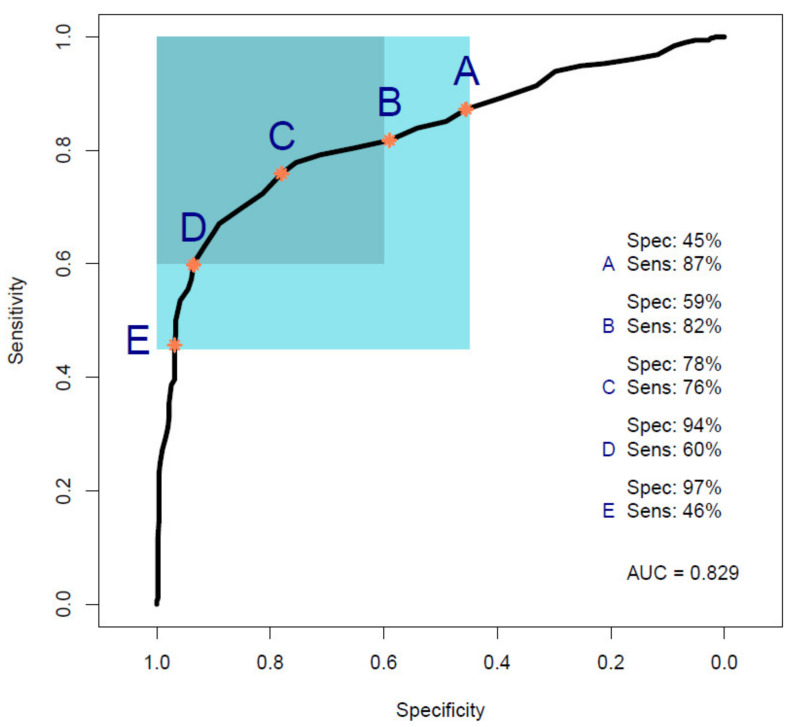
Receiver operating characteristic (ROC) curve analysis calculated from a partial least squares discriminant analysis (PLS-DA) model performed on Cohort B. Point A shows the point of maximum sensitivity and point E shows the point of maximum specificity when the model is tuned to give a specificity or sensitivity of 45%, respectively, as highlighted by the light blue square. Point B shows the point of maximum sensitivity and point D shows the point of maximum specificity when the model is tuned to give a specificity or sensitivity of 60%, respectively, as highlighted by the darker light blue square. Point C represents the balance point between sensitivity and specificity, whilst remaining in the target regions (Sens, sensitivity; Spec, specificity; AUC, area under the ROC curve).

**Figure 5:**
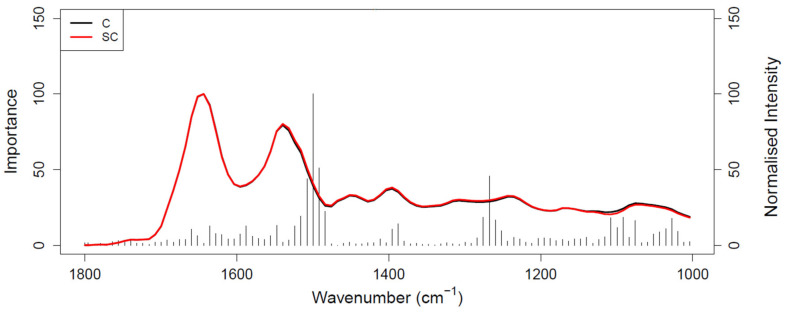
Gini importance plot of RF analysis performed on Cohort B; C (black) represents the pancreatic cancer spectrum and SC (red) represents the symptomatic control spectrum.

**Figure 6:**
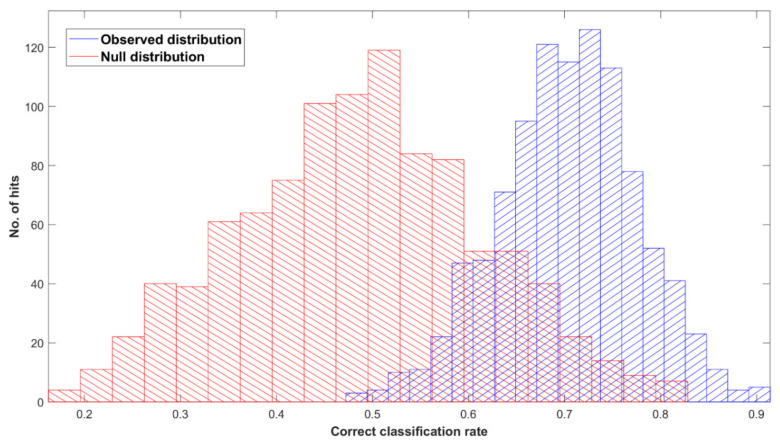
Permutation tests’ output plot; null (red) and observed (blue) distribution classification rated for Cohort B with a partial least squares discriminant analysis (PLS-DA) classification model after 1000 bootstraps, using pre-processed spectra.

Figure 4 shows the ROC curve calculated from PLS-DA (Table 3), discriminating between 35 pancreatic cancer and 35 symptomatic control samples. The 35 cancer samples in Cohort B were selected from the 100 cancer samples in Cohort A by age- and sex-matching the 35 symptomatic controls included in Cohort B. The ROC curve shows a balanced sensitivity and specificity point of 76% and 78%, respectively; however, despite the more challenging classification, as symptomatic control samples consist of patients presenting similar symptoms as cancerous patients—essentially comorbidities and/or confounding diseases—the AUC was equal to 0.829, which still indicates a high level of diagnostic separability [37]. Moreover, a maximum sensitivity of 82% and maximum specificity of 94% were obtained when the classification threshold was tuned to give specificity or sensitivity of 60%, respectively. When changing the thresholds to give specificity or sensitivity of 45% (light blue square), the sensitivity and specificity increased further, to 87% and 97%, respectively; these results highlight the potential of the technique to be adapted to the international diagnostic markets, in scenarios where sensitivity may be preferred to specificity, and vice versa [12]. RF, PLS-DA, and SVM performed on Cohort B obtained overall sensitivity ranging between 69.5 ± 14.3% and 72.0 ± 13.1%, and specificity between 72.7 ± 16.0% and 85.1 ± 12.9%, with an accuracy between 72.3 ± 8.2% and 77.5 ± 10.1% (Table 3). The higher variation (i.e., standard deviation (SD)) seen in this cohort, when compared to the SD values of Cohort A (Table 2), was most likely related to the lower number of samples present. RF was seen to be the best performer in terms of sensitivity, PLS-DA was the more specific method, and SVM had the better overall accuracy (Table 3). As seen for the AUC value, the reduction in sensitivity and specificity (compared to Cohort A) was expected due to the examination of symptomatic controls, albeit describing a more real-world scenario. The RF Gini importance plot for Cohort B (Figure 5) highlighted more important features than those seen in Cohort A’s Gini plot (Figure 2) [33,41]. A table containing the top 15 wavenumbers in order of importance, with corresponding tentative spectral assignments, is reported in the Supplementary Materials (Table S6) [22,42,43,44]. Pancreatic cancers and symptomatic controls seemed to be mostly distinguished on the right-hand side of the amide II peak (around 1500 cm^−1^, δ(NH)/ν(CN)), across the asymmetric stretching of PO_2−_ (around 1270 cm^−1^) and the symmetric stretching of the CO-O-C bond in carbohydrates (between 1000 and 1100 cm^−1^). Similar discriminating spectral characteristics—in particular concerning the amide II region—have already been proven to be key features in the differentiation between brain tumors and healthy controls [22,33,44] although, to our knowledge, there are no publications to date that explore the impact of symptomatic patients on the spectral discrimination from cancerous patients using the RF algorithm. The permutation test showed a partial overlap of the null and observed distributions (Figure 6), although with a *p*-value of 0.0500 still proving the statistical significance of the classification of the pancreatic cancers and symptomatic controls [39].

#### 3.2.2. Cohort C

A greater number of cancer samples (*n* = 100) was introduced in Cohort C to investigate the impact of a class imbalance on the machine learning algorithms’ performance, with the positive class (cancer) containing more patients than the negative class (symptomatic, *n* = 35). SMOTE was applied during the data analysis to reduce bias during model training by artificially mixing the data in order to create “new” samples [22]. Figure S1 shows the ROC curve obtained from the PLS-DA model. The analysis performed similarly in terms of results, with a balanced sensitivity and specificity point of 79% and 77%, respectively (3% higher in sensitivity and 1% lower in specificity compared to Cohort B’s ROC analysis; Figure 4), and an AUC of 0.849 (0.02 higher than AUC of Cohort B’s ROC curve). The sensitivity values improved, increasing to 87.4 ± 6.4%; however, the specificity dropped by 21.3% in the RF analysis and 6.0% in SVM; PLS-DA did not respect the trend, presenting an increase in both sensitivity and specificity, of 2.9 and 2.1%, respectively. The loss of specificity for both the RF and SVM models, and the opposite behavior of the PLS-DA model, show how a strong class imbalance can still strongly impact the outcomes, even if using sampling methods to overcome the bias in the classification. The classifications’ outputs can be seen in Table S7. The permutation test confirmed the overall worsening impact of the class imbalance on the classification outcomes, showing an overlap of the null and observed distributions (Figure S2), and returning a *p*-value of 0.31. Even though the machine learning algorithms and ROC analysis showed a high degree of diagnostic discrimination, further analyses with additional symptomatic patients and larger cohorts are required to ensure reliable, robust, and significant results in the intended-use population.

## 4. Discussion

Pancreatic cancer seems to have only recently been investigated by spectroscopic researchers; thus, there are few publications highlighting the potential of alternative diagnostic techniques to detect this type of cancer in its early stages using non-invasive methods [16]. Surface-enhanced Raman spectroscopy (SERS) has been used to detect pancreatic tumor biomarkers in blood, with promising results, but only small cohorts of patients in each group (*n* = 5) have been employed [45,46]. In 2018, Habartovà et al. investigated the diagnostic performance of both Raman and ATR-FTIR spectroscopy using blood plasma; linear discriminant analysis (LDA) had a sensitivity of 91% and a specificity of 74% for Raman, whilst it had a sensitivity of 76% and specificity of 48% for ATR-FTIR, with an overall accuracy of 84% (Raman) and 65% (ATR-FTIR), in discriminating between cancer and healthy control samples [47]; moreover, ROC analysis produced AUC values of 0.944 for Raman and 0.747 for ATR-FTIR, as measures of the diagnostic ability of the models to discern cancer from healthy controls [47]. However, small cohorts of samples consisting of 34 pancreatic cancer and 23 (*n* < 25) healthy control samples were used to perform their analysis, validating the results with the leave-one-out cross-validation process only [47]; moreover, the lack of symptomatic control samples does not allow an accurate representation of reality, where patients referred for cancer screening are not healthy people but those presenting with suspicious symptomatology.

According to the NICE guidelines, after suspecting a pancreatic tumor due to a CA 19-9 test and abdominal imaging, the next point of call consists of a multiphase pancreatic protocol computed tomography (CT) with intravenous contrast, followed by fluorodeoxyglucose-positron emission tomography (FDG-PET) coupled with CT and/or endoscopic ultrasound (EUS) with biopsy [48]. Combining the NHS reference costs and the SYMPTOM study numbers in a health economic model, it has been calculated that if every patient with suspected pancreatic cancer undergoes the full diagnostic pathway according to the NICE guidelines, the NHS would use over GBP 11 million to run an estimate of 16,800 medical tests for the diagnosis of pancreatic cancer in the UK, detecting only about 5000 cases per year [48,49,50,51]. The employment of ATR-FTIR spectroscopy as an alternative diagnostic method in the clinical setting could save over GBP 10 million in NHS resources [51].

This proof-of-concept study examining the ability of ATR-FTIR serum spectroscopy to diagnose pancreatic cancer illustrates the outstanding potential of the technique by achieving promising statistical metric outputs, when differentiating between 100 cancer and 100 healthy control samples using three different machine learning algorithms. The best-performing model was the PLS-DA method, which reported values of up to 91.7 ± 5.4% sensitivity, 87.7 ± 4.8% specificity, and accuracy of 89.7 ± 2.9% (Table 2). In addition, ROC analysis (Figure 1) gave an AUC of 0.954, proving an excellent degree of diagnostic discrimination. The statistical significance was calculated to be optimal, based on a PLS-DA model after 1000 bootstraps with a *p*-value < 0.0001 (Figure 3).

Moreover, the potential to discriminate between 35 cancer and 35 symptomatic control samples (i.e., people presenting symptoms of pancreatic cancer and recruited, but then diagnosed as non-cancerous patients, with comorbidities) was investigated, giving an AUC of 0.829 using PLS-DA, which at 70% sensitivity gives 85% specificity (PLS-DA; Table 3 and Figure 4). Notwithstanding the fact that Cohort B was smaller than the cohort with healthy control samples (Cohort A), respectable classification outputs were achieved, which were also proven to be statistically significant through bootstrapping validation (Figure 6).

Further analysis of 100 cancer and 35 symptomatic control samples was performed to understand the impact of the number of samples and class imbalance on the machine learning algorithms’ classifications. Even though ROC analysis resulted in a better AUC of 0.849 when using the SMOTE sampling method to compensate for the class imbalance and avoid bias in the classifications, the outputs were not statistically significant (Figure S2), and were clearly affected by the class imbalance, losing specificity with the RF and SVM methods, despite a good increase in the sensitivity values (Table S7 and Figure S1).

Despite the great outputs reported in this proof-of-concept study, there are still additional research queries that need to be addressed in order to prove the utility of this spectroscopic liquid biopsy in the current clinical setting. The test has shown its ability to discern between cancer and healthy/symptomatic control samples; however, it requires further testing in a wider scenario, to include subsets of different types and stages of pancreatic cancer, in order to address the need for earlier detection. Furthermore, this proof-of-concept study included samples from both prospective recruitments and biobanks; an extensive prospective clinical trial is imperative to test the actual potential of this technique in the pancreatic cancer setting, as this would ultimately provide the broad range of tumor types, stages/grades, and symptomatic controls needed to reflect the current pancreatic cancer burden. Moreover, investigating the impact of comorbidities or localized pathologies—such as chronic pancreatitis, the presence of gallbladder stones, and/or other tumors—should be considered as a future step to address the impact that an additional inflammatory condition has on the accuracy of the test, and the ability of the test to discern between pancreatic cancer, other benign pathologies, and different types of tumor with similar symptomology (e.g., gastric, colorectal, ovarian, etc.).

Nonetheless, the analysis of human blood serum with ATR-FTIR spectroscopy has the potential to represent a noteworthy addition and future alternative to the current diagnostic pathway for pancreatic cancer, as well as to screening programs for high-risk patient groups (e.g., based on family history), thanks to its simple use, minimal invasiveness and, as shown in this study, high diagnostic performance in distinguishing between cancer and healthy control patients. Additionally, the ability of achieving such promising results in discriminating cancer and symptomatic control patients with a smaller cohort of patients (*n* = 70) has paved the way for future research with a greater number of samples for both classes, which would likely provide more robust and reliable results.

## 5. Conclusions

Alternative methods to detect pancreatic cancer are urgently needed in the clinical environment to enable earlier detection and, thus, effective therapies and/or surgical treatments to improve prognosis and increase survival rates.

This study demonstrates the ability of our novel liquid biopsy approach, using ATR-FTIR spectroscopy on dried blood serum, to discriminate between cancer and healthy control samples, with sensitivity of 92%, specificity of 88%, and AUC = 0.95 (PLS-DA). In addition, our test can identify cancerous patients among symptomatic patients with 70% sensitivity, 85% specificity, and AUC = 0.83 (PLS-DA).

ATR-FTIR spectroscopy has been demonstrated to have exceptional potential to become a reliable tool in the clinical diagnosis of pancreatic cancer, as an aid to obtain earlier detection and better quality of life of patients.

## Figures and Tables

**Table 1:** Samples and patient details of the cohorts analyzed. (F, females; M, males).

Cohort	Samples	Average Age (Years)	Sex (%)
A	100 cancers	63	F = 42; M = 58
	100 healthy controls	65	F = 43; M = 57
B	35 cancers ^†^	63	F = 46; M = 54
	35 symptomatic controls	63	F = 46; M = 54
C	100 cancers	63	F = 42; M = 58
	35 symptomatic controls	63	F = 46; M = 54

^†^ The 35 cancer samples in cohort B were selected from the 100 cancer samples in cohort A by age- and sex-matching the 35 symptomatic controls included in cohort B.

**Table 2:** Statistical performances of random forest (RF), partial least squares discriminant analysis (PLS-DA), support-vector machine (SVM), and receiver operating characteristic (ROC) analysis performed on Cohort A (AUC, area under the ROC curve; SD, standard deviation).

Cohort A	RF			PLS-DA			SVM		
Sensitivity ± SD (%)	84.4	±	7.2	91.7	±	5.4	89.3	±	6.0
Specificity ± SD (%)	86.3	±	7.1	87.7	±	4.8	87.8	±	6.2
Accuracy ± SD (%)	85.4	±	4.5	89.7	±	2.9	88.5	±	4.3
ROC (AUC)	0.905			0.954			0.946		

**Table 3:** Statistical performances of random forest (RF), partial least squares discriminant analysis (PLS-DA), support-vector machine (SVM), and receiver operating characteristic (ROC) analysis performed on Cohort B (AUC, area under the ROC curve; SD, standard deviation).

Cohort B	RF			PLS-DA			SVM			
Sensitivity ± SD (%)	72.0	±	13.1	69.8	±	14.3	71.6	±	15.9	
Specificity ± SD (%)	72.7	±	16.0	85.1	±	12.9	83.3	±	14.9	
Accuracy ± SD (%)	72.3	±	8.9	76.5	±	7.8	77.5	±	10.1	
ROC (AUC)	0.809			0.829			0.793			

## Data Availability

All data are presented within the manuscript or in the Supplementary Materials. For any further information, contact the corresponding author.

## Supplementary Materials

The following supporting information can be downloaded at https://www.mdpi.com/article/10.3390/cancers14133048/s1, Table S1: Cohort A; patients’ information. Sorted by Age column (M, males; F, females; T.S., Tissue Solutions; B.C.C., Beatson Cancer Centre; S.H., Singleton Hospital; G.H., Gloucestershire Hospital), Table S2: Cohort B; patients’ information. Sorted by Age column (M, males; F, females; T.S., Tissue Solutions; B.C.C., Beatson Cancer Centre; S.H., Singleton Hospital; G.H., Gloucestershire Hospital), Table S3: Cohort C; patients’ information. Sorted by Age column (M, males; F, females; T.S., Tissue Solutions; B.C.C., Beatson Cancer Centre; S.H., Singleton Hospital; G.H., Gloucestershire Hospital), Table S4: Summary table on age and sex information about all of the classes used to perform the analysis (F, females; M, males), Table S5: Top 15 wavenumbers from random forest (RF) classification of Cohort A, with tentative assignments (where νs = symmetrical stretching, νas = asymmetrical stretching, and δ = bending), Table S6: Top 15 wavenumbers from random forest (RF) classification of Cohort B, with tentative assignments (where νs = symmetrical stretching, νas = asymmetrical stretching, and δ = bending), Table S7: Statistical performances of random forest (RF), partial least squares discriminant analysis (PLS-DA), support-vector machine (SVM), and receiver operating characteristic (ROC) analysis performed on Cohort C (AUC, area under the ROC curve; SD, standard deviation), Figure S1: Receiver operating characteristic (ROC) curve analysis calculated from a partial least squares discriminant analysis (PLS-DA) model performed on Cohort C. The colored box indicates the region with sensitivity and specificity greater than 60%. The orange dots indicate the points within that region that have maximum sensitivity (A), maximum specificity (B), and balanced sensitivity and specificity (C) (Sens, sensitivity; Spec, specificity; AUC, area under the ROC curve), Figure S2: Permutation tests’ output plot; null (red) and observed (blue) distribution classification rated for Cohort C with a partial least squares-discriminant analysis (PLS-DA) classification model after 1000 bootstraps, using pre-processed spectra.
