# Supplementary material for: Liquid Biopsy for Pancreatic Cancer Detection Using Infrared Spectroscopy
Source: Cancers (Basel). 2022 Jun 21;14(13):3048. doi: 10.3390/cancers14133048 (PMC9264892; doi:10.3390/cancers14133048)
Supplement: Supplementary file 1 [file cancers-14-03048-s001.zip › cancers-1746221-supplementary.pdf]

cancers

MDPI

Supplementary Information

Liquid Biopsy for Pancreatic Cancer Detection Using Infrared Spectroscopy

Alexandra Sala [1,2], James M. Cameron [2], Cerys A. Jenkins [3], Hugh Barr [4], Loren Christie [1,2], Justin J. A. Conn [2], Thomas R. Jeffry Evans [5], Dean A. Harris [6], David S. Palmer [1,2], Christopher Rinaldi [7], Ashton G. Theakstone [7] and Matthew J. Baker [2,*]

[1] Department of Pure and Applied Chemistry, University of Strathclyde, Thomas Graham Building, Glasgow G1 1XL, UK; alexandra.sala@dxcover.com (A.S.); loren.christie@dxcover.com (L.C.); david.palmer@dxcover.com (D.S.P.)
[2] Dxcover Limited, Royal College Building, Glasgow G1 1XW, UK; james.cameron@dxcover.com (J.M.C.); justin.conn@dxcover.com (J.J.A.C.)
[3] Swansea University Medical School, Swansea University, Swansea SA2 8PP, UK; cerys.jenkins@cansenseltd.com
[4] Gloucestershire Hospitals NHS Foundation Trust, Gloucester GL1 2EL, UK; hugh.barr@nhs.net
[5] Institute of Cancer Sciences, University of Glasgow, Glasgow G61 1BD, UK; j.evans@beatson.gla.ac.uk
[6] Swansea Bay University Local Health Board, Singleton Hospital, Swansea SA2 8QA, UK; dean.a.harris@wales.nhs.uk
[7] Department of Pure and Applied Chemistry, University of Strathclyde, The Technology and Innovation Centre, Glasgow G1 1RD, UK; christopher.rinaldi@aureumdx.com (C.R.); ashton.theakstone@strath.ac.uk (A.G.T.)
* Correspondence: matthew.baker@dxcover.com

Supplementary Text

Model Hyperparameters

Model hyperparameters were tuned using nested-cross-validation as described in the manuscript.

The hyperparameters that were optimized for each model were:

- For random forest (RF), the parameter identifying the number of randomly selected predictors used to fit each tree (mtry) was set to 28, corresponding to the square root of the number of features contained in the region examined.
- For partial least squares - discriminant analysis (PLS-DA), the parameter controlling the number of components selected to build the analysis (ncomp) was selected to pick the best number of components between 1 and 30.
- For support vector machine (SVM), the optimal value of the cost parameter was selected from values between 0.01 to 0.1 with an interval of 0.01.

Table S1. Cohort A; patients' information. Sorted by Age column. (M, males; F, females; T.S., Tissue Solutions; B.C.C., Beatson Cancer Centre; S.H., Singleton Hospital; G.H., Gloucestershire Hospital).

ID	Sex	Age	Class	Diagnosis	Origin
D308355	M	30	Control	N/A	T.S. Ltd. (Glasgow)
BH363	F	40	Cancer	Adenocarcinoma	B.C.C. (Glasgow)
PC9	F	45	Cancer	Adenocarcinoma	T.S. Ltd. (Glasgow)
R527002	F	46	Control	N/A	T.S. Ltd. (Glasgow)
PC10	F	48	Cancer	Adenocarcinoma	T.S. Ltd. (Glasgow)
PC24	F	48	Cancer	Adenocarcinoma	T.S. Ltd. (Glasgow)
PC1	M	50	Cancer	Adenocarcinoma	T.S. Ltd. (Glasgow)
PC19	M	50	Cancer	Adenocarcinoma	T.S. Ltd. (Glasgow)
PC23	F	50	Cancer	Adenocarcinoma	T.S. Ltd. (Glasgow)
BH354	F	51	Cancer	Met Adenocarcinoma	B.C.C. (Glasgow)
SH086	M	51	Cancer	Met Adenocarcinoma	S.H. (Swansea)
PC11	M	51	Cancer	Adenocarcinoma	T.S. Ltd. (Glasgow)
PC22	F	51	Cancer	Adenocarcinoma	T.S. Ltd. (Glasgow)
PC29	F	51	Cancer	Adenocarcinoma	T.S. Ltd. (Glasgow)
R527163	M	51	Control	N/A	T.S. Ltd. (Glasgow)
D308509	M	52	Control	N/A	T.S. Ltd. (Glasgow)
R527087	F	52	Control	N/A	T.S. Ltd. (Glasgow)
R527545	M	52	Control	N/A	T.S. Ltd. (Glasgow)
125254691606	M	53	Cancer	Met Adenocarcinoma	S.H. (Swansea)
125262256606	M	53	Cancer	Adenocarcinoma	S.H. (Swansea)
D308477	F	53	Control	N/A	T.S. Ltd. (Glasgow)
SH079	M	53	Cancer	Adenocarcinoma	S.H. (Swansea)
PC15	M	53	Cancer	Adenocarcinoma	T.S. Ltd. (Glasgow)
PC7	F	53	Cancer	Adenocarcinoma	T.S. Ltd. (Glasgow)
R526955	M	53	Control	N/A	T.S. Ltd. (Glasgow)
R527032	M	53	Control	N/A	T.S. Ltd. (Glasgow)
R527557	M	53	Control	N/A	T.S. Ltd. (Glasgow)
R527751	F	53	Control	N/A	T.S. Ltd. (Glasgow)
PC12	F	54	Cancer	Adenocarcinoma	T.S. Ltd. (Glasgow)
PC2	M	54	Cancer	Adenocarcinoma	T.S. Ltd. (Glasgow)
PC30	M	54	Cancer	Adenocarcinoma	T.S. Ltd. (Glasgow)
R527067	F	54	Control	N/A	T.S. Ltd. (Glasgow)
R527772	M	54	Control	N/A	T.S. Ltd. (Glasgow)
R527859	M	54	Control	N/A	T.S. Ltd. (Glasgow)
P128032	M	55	Cancer	Adenocarcinoma	T.S. Ltd. (Glasgow)
BH368	M	55	Cancer	Adenocarcinoma	B.C.C. (Glasgow)
PC31	M	55	Cancer	Adenocarcinoma	T.S. Ltd. (Glasgow)
PC6	M	55	Cancer	Adenocarcinoma	T.S. Ltd. (Glasgow)
R526928	M	55	Control	N/A	T.S. Ltd. (Glasgow)
R527713	M	55	Control	N/A	T.S. Ltd. (Glasgow)
R527868	M	55	Control	N/A	T.S. Ltd. (Glasgow)
P128011	M	55	Cancer	Adenocarcinoma	T.S. Ltd. (Glasgow)
125310511306	M	56	Cancer	Adenocarcinoma	S.H. (Swansea)
SH078	M	56	Cancer	Adenocarcinoma	S.H. (Swansea)
PC16	F	56	Cancer	Adenocarcinoma	T.S. Ltd. (Glasgow)

Table S1 (cont.)

ID	Sex	Age	Class	Diagnosis	Origin
PC25	M	56	Cancer	Adenocarcinoma	T.S. Ltd. (Glasgow)
R526967	F	56	Control	N/A	T.S. Ltd. (Glasgow)
R527272	M	56	Control	N/A	T.S. Ltd. (Glasgow)
R527768	M	56	Control	N/A	T.S. Ltd. (Glasgow)
R527771	M	56	Control	N/A	T.S. Ltd. (Glasgow)
BH362	F	57	Cancer	Adenocarcinoma	B.C.C. (Glasgow)
BH367	F	57	Cancer	Adenocarcinoma	B.C.C. (Glasgow)
125270584106	M	57	Cancer	Adenocarcinoma	S.H. (Swansea)
SH261	M	57	Cancer	NET	S.H. (Swansea)
TJ230118	F	57	Cancer	Met (Peritoneal) Carcinoma	S.H. (Swansea)
R527707	M	57	Control	N/A	T.S. Ltd. (Glasgow)
R527803	F	57	Control	N/A	T.S. Ltd. (Glasgow)
R527864	M	57	Control	N/A	T.S. Ltd. (Glasgow)
BH373	F	58	Cancer	Adenocarcinoma	B.C.C. (Glasgow)
PC8	M	58	Cancer	Adenocarcinoma	T.S. Ltd. (Glasgow)
A201875	M	58	Control	N/A	T.S. Ltd. (Glasgow)
R527170	M	58	Control	N/A	T.S. Ltd. (Glasgow)
PC26	M	59	Cancer	Adenocarcinoma	T.S. Ltd. (Glasgow)
R527622	M	59	Control	N/A	T.S. Ltd. (Glasgow)
R527872	M	59	Control	N/A	T.S. Ltd. (Glasgow)
PC13	M	60	Cancer	Adenocarcinoma	T.S. Ltd. (Glasgow)
PC28	M	60	Cancer	Adenocarcinoma	T.S. Ltd. (Glasgow)
PC3	F	60	Cancer	Adenocarcinoma	T.S. Ltd. (Glasgow)
R527061	M	60	Control	N/A	T.S. Ltd. (Glasgow)
R527445	F	60	Control	N/A	T.S. Ltd. (Glasgow)
R527730	M	60	Control	N/A	T.S. Ltd. (Glasgow)
P128002	F	60	Cancer	Adenocarcinoma	T.S. Ltd. (Glasgow)
P128010	F	61	Cancer	Adenocarcinoma	T.S. Ltd. (Glasgow)
SH328	F	61	Cancer	Adenocarcinoma	S.H. (Swansea)
PC4	M	61	Cancer	Adenocarcinoma	T.S. Ltd. (Glasgow)
R526980	M	61	Control	N/A	T.S. Ltd. (Glasgow)
R527027	M	61	Control	N/A	T.S. Ltd. (Glasgow)
R527854	M	61	Control	N/A	T.S. Ltd. (Glasgow)
BH401	M	62	Cancer	Adenocarcinoma	B.C.C. (Glasgow)
PC14	M	62	Cancer	Adenocarcinoma	T.S. Ltd. (Glasgow)
PC17	F	62	Cancer	Adenocarcinoma	T.S. Ltd. (Glasgow)
R527078	M	62	Control	N/A	T.S. Ltd. (Glasgow)
R527614	M	62	Control	N/A	T.S. Ltd. (Glasgow)
R527818	M	62	Control	N/A	T.S. Ltd. (Glasgow)
1252886441	F	63	Cancer	Met Adenocarcinoma	S.H. (Swansea)
SH304	F	63	Cancer	Adenocarcinoma	S.H. (Swansea)
R527004	M	63	Control	N/A	T.S. Ltd. (Glasgow)
R527862	M	63	Control	N/A	T.S. Ltd. (Glasgow)
BH416	M	64	Cancer	Met Adenocarcinoma	B.C.C. (Glasgow)
BH445	F	64	Cancer	Adenocarcinoma	B.C.C. (Glasgow)
PC27	F	64	Cancer	Adenocarcinoma	T.S. Ltd. (Glasgow)
PC32	F	64	Cancer	Adenocarcinoma	T.S. Ltd. (Glasgow)

Table S1 (cont.)

ID	Sex	Age	Class	Diagnosis	Origin
PC5	M	64	Cancer	Adenocarcinoma	T.S. Ltd. (Glasgow)
BH418	F	65	Cancer	Adenocarcinoma	B.C.C. (Glasgow)
SH085	F	65	Cancer	Adenocarcinoma	S.H. (Swansea)
PC20	F	65	Cancer	Adenocarcinoma	T.S. Ltd. (Glasgow)
A201896	M	65	Control	N/A	T.S. Ltd. (Glasgow)
KO50	F	65	Control	N/A	T.S. Ltd. (Glasgow)
KO51	F	65	Control	N/A	T.S. Ltd. (Glasgow)
KO52	F	65	Control	N/A	T.S. Ltd. (Glasgow)
ST115	M	65	Control	Dilatational cardiomyopathy	T.S. Ltd. (Glasgow)
ST156	M	65	Control	N/A	T.S. Ltd. (Glasgow)
ST81	M	65	Control	Angina pectoris	T.S. Ltd. (Glasgow)
BH353	F	66	Cancer	Adenocarcinoma	B.C.C. (Glasgow)
SH309	F	66	Cancer	NET	S.H. (Swansea)
PC18	M	66	Cancer	Adenocarcinoma	T.S. Ltd. (Glasgow)
PC21	M	66	Cancer	Adenocarcinoma	T.S. Ltd. (Glasgow)
KO140	F	66	Control	N/A	T.S. Ltd. (Glasgow)
ST38	F	66	Control	N/A	T.S. Ltd. (Glasgow)
ST39	F	66	Control	Diaphragmatic hernia	T.S. Ltd. (Glasgow)
ST64	M	66	Control	Angina pectoris	T.S. Ltd. (Glasgow)
BH372	M	67	Cancer	Adenocarcinoma	B.C.C. (Glasgow)
BH422	M	67	Cancer	Adenocarcinoma	B.C.C. (Glasgow)
BH448	M	67	Cancer	Adenocarcinoma	B.C.C. (Glasgow)
1252672929	M	67	Cancer	Adenocarcinoma	S.H. (Swansea)
KO124	F	67	Control	N/A	T.S. Ltd. (Glasgow)
KO139	F	67	Control	N/A	T.S. Ltd. (Glasgow)
KO144	M	67	Control	N/A	T.S. Ltd. (Glasgow)
ST149	F	67	Control	N/A	T.S. Ltd. (Glasgow)
ST153	M	67	Control	N/A	T.S. Ltd. (Glasgow)
ST160	F	67	Control	N/A	T.S. Ltd. (Glasgow)
BH350	F	68	Cancer	Adenocarcinoma	B.C.C. (Glasgow)
125289145806	M	68	Cancer	NET	S.H. (Swansea)
ST108	M	68	Control	Angina pectoris	T.S. Ltd. (Glasgow)
ST109	F	68	Control	Angina pectoris	T.S. Ltd. (Glasgow)
ST132	F	68	Control	Angina pectoris	T.S. Ltd. (Glasgow)
ST150	F	68	Control	N/A	T.S. Ltd. (Glasgow)
ST44	M	68	Control	Angina pectoris	T.S. Ltd. (Glasgow)
ST49	M	68	Control	COPD (lungs disease)	T.S. Ltd. (Glasgow)
BH403	M	69	Cancer	Met Adenocarcinoma	B.C.C. (Glasgow)
1252640380	M	69	Cancer	Liposarcoma	S.H. (Swansea)
1252899776	F	69	Cancer	Adenocarcinoma	S.H. (Swansea)
ST120	F	69	Control	LPFB (heart disease)	T.S. Ltd. (Glasgow)
125315237106	M	70	Cancer	Invasive IPMN	S.H. (Swansea)
SH077	M	70	Cancer	Adenocarcinoma	S.H. (Swansea)
KO142	M	70	Control	N/A	T.S. Ltd. (Glasgow)
ST118	F	70	Control	Atherosclerosis	T.S. Ltd. (Glasgow)
ST46	M	70	Control	Angina pectoris	T.S. Ltd. (Glasgow)
ST67	F	70	Control	Aortic stenosis	T.S. Ltd. (Glasgow)

Table S1 (cont.)

ID	Sex	Age	Class	Diagnosis	Origin
BH413	F	71	Cancer	Met Adenocarcinoma	B.C.C. (Glasgow)
OD211217	M	71	Cancer	Met Adenocarcinoma	S.H. (Swansea)
P128017	M	71	Cancer	Adenocarcinoma	T.S. Ltd. (Glasgow)
KO128	F	71	Control	N/A	T.S. Ltd. (Glasgow)
KO147	M	71	Control	N/A	T.S. Ltd. (Glasgow)
KO148	M	71	Control	N/A	T.S. Ltd. (Glasgow)
ST42	F	71	Control	COPD (lungs disease)	T.S. Ltd. (Glasgow)
ST88	M	71	Control	Atherosclerosis	T.S. Ltd. (Glasgow)
BH406	M	72	Cancer	Met Adenocarcinoma	B.C.C. (Glasgow)
BH414	F	72	Cancer	Adenocarcinoma	B.C.C. (Glasgow)
KO143	M	72	Control	N/A	T.S. Ltd. (Glasgow)
ST105	M	72	Control	Atherosclerosis	T.S. Ltd. (Glasgow)
ST129	M	72	Control	Myocardial infarction	T.S. Ltd. (Glasgow)
ST91	M	72	Control	Angina pectoris	T.S. Ltd. (Glasgow)
1252813381	M	73	Cancer	Adenocarcinoma	S.H. (Swansea)
SH225	M	73	Cancer	NET	S.H. (Swansea)
SH269	M	73	Cancer	NET	S.H. (Swansea)
SH314	M	73	Cancer	Invasive IPMN	S.H. (Swansea)
1252409748	M	74	Cancer	Adenocarcinoma	S.H. (Swansea)
SH276	F	74	Cancer	Adenocarcinoma	S.H. (Swansea)
ST106	F	74	Control	N/A	T.S. Ltd. (Glasgow)
ST137	F	74	Control	N/A	T.S. Ltd. (Glasgow)
ST158	F	74	Control	N/A	T.S. Ltd. (Glasgow)
125312955206	M	75	Cancer	NET	S.H. (Swansea)
RT101117	M	75	Cancer	Adenocarcinoma	S.H. (Swansea)
SH313	M	75	Cancer	Adenocarcinoma	S.H. (Swansea)
SH320	F	75	Cancer	Met Adenocarcinoma	S.H. (Swansea)
SH322	M	75	Cancer	Met Adenocarcinoma	S.H. (Swansea)
SH330	M	75	Cancer	Adenocarcinoma	S.H. (Swansea)
GH69	M	75	Control	Cardiovascular diseases	T.S. Ltd. (Glasgow)
KO122	F	75	Control	N/A	T.S. Ltd. (Glasgow)
ST107	M	75	Control	Myocardial infarction	T.S. Ltd. (Glasgow)
ST116	F	75	Control	Myocardial infarction	T.S. Ltd. (Glasgow)
ST131	F	75	Control	Myocardial infarction	T.S. Ltd. (Glasgow)
ST152	F	75	Control	N/A	T.S. Ltd. (Glasgow)
ST157	F	75	Control	N/A	T.S. Ltd. (Glasgow)
ST66	M	75	Control	IHD (cardiosclerosis)	T.S. Ltd. (Glasgow)
BH361	M	76	Cancer	Met Adenocarcinoma	B.C.C. (Glasgow)
BH434	F	76	Cancer	Met Adenocarcinoma	B.C.C. (Glasgow)
CT091117	F	76	Cancer	Met Adenocarcinoma	S.H. (Swansea)
SH065	F	76	Cancer	Adenocarcinoma	S.H. (Swansea)
SH203	F	76	Cancer	Adenocarcinoma	S.H. (Swansea)
ST86	F	76	Control	Mycotic pneumonia	T.S. Ltd. (Glasgow)
ST134	M	77	Control	Angina pectoris	T.S. Ltd. (Glasgow)
ST40	F	78	Control	Diaphragmatic hernia	T.S. Ltd. (Glasgow)
ST47	F	78	Control	Thyroiditis + Angina pectoris	T.S. Ltd. (Glasgow)
ST100	M	79	Control	Myocardial infarction	T.S. Ltd. (Glasgow)

Table S1 (cont.)

ID	Sex	Age	Class	Diagnosis	Origin
ST113	F	79	Control	N/A	T.S. Ltd. (Glasgow)
ST114	F	79	Control	Abdominal hernia	T.S. Ltd. (Glasgow)
ST130	F	79	Control	Sick sinus syndrome	T.S. Ltd. (Glasgow)
ST133	M	79	Control	N/A	T.S. Ltd. (Glasgow)
ST135	F	79	Control	Myocardial infarction	T.S. Ltd. (Glasgow)
1252341008	M	80	Cancer	Adenocarcinoma	S.H. (Swansea)
1252637561	F	80	Cancer	Met Adenocarcinoma	S.H. (Swansea)
SH158	M	80	Cancer	Adenocarcinoma	S.H. (Swansea)
KO145	F	80	Control	N/A	T.S. Ltd. (Glasgow)
ST103	M	80	Control	Macular degeneration	T.S. Ltd. (Glasgow)
ST85	F	80	Control	Myocardial infarction	T.S. Ltd. (Glasgow)
1252812461	F	83	Cancer	Adenocarcinoma	S.H. (Swansea)
125234430306	M	83	Cancer	Met Adenocarcinoma	S.H. (Swansea)
G004	M	87	Cancer	Adenocarcinoma	G.H. (Gloucester)

Table S2. Cohort B; patients' information. Sorted by Age column. (M, males; F, females; T.S., Tissue Solutions; B.C.C., Beatson Cancer Centre; S.H., Singleton Hospital; G.H., Gloucestershire Hospital).

ID	Sex	Age	Class	Diagnosis	Origin
1252408663	M	28	Symptom. Control	N/A	S.H. (Swansea)
DOJ101117	M	36	Symptom. Control	Ulcerative colitis	S.H. (Swansea)
KS120118	F	42	Symptom. Control	Colorectal polyp	S.H. (Swansea)
SH302	F	42	Symptom. Control	IBS	S.H. (Swansea)
TJ090218	M	42	Symptom. Control	Colorectal polyp	S.H. (Swansea)
PC9	F	45	Cancer	Adenocarcinoma	T.S. Ltd. (Glasgow)
PC10	F	48	Cancer	Adenocarcinoma	T.S. Ltd. (Glasgow)
PC19	M	50	Cancer	Adenocarcinoma	T.S. Ltd. (Glasgow)
SH149	M	51	Symptom. Control	N/A	S.H. (Swansea)
SH144	M	52	Symptom. Control	N/A	S.H. (Swansea)
125254691606	M	53	Cancer	Met Adenocarcinoma	S.H. (Swansea)
125262256606	M	53	Cancer	Adenocarcinoma	S.H. (Swansea)
PC7	F	53	Cancer	Adenocarcinoma	T.S. Ltd. (Glasgow)
PC12	F	54	Cancer	Adenocarcinoma	T.S. Ltd. (Glasgow)
PC2	M	54	Cancer	Adenocarcinoma	T.S. Ltd. (Glasgow)
BH368	M	55	Cancer	Adenocarcinoma	B.C.C. (Glasgow)
SL260118	M	55	Symptom. Control	Polyp surveillance	S.H. (Swansea)
125310511306	M	56	Cancer	Adenocarcinoma	S.H. (Swansea)
SH078	M	56	Cancer	Adenocarcinoma	S.H. (Swansea)
PC16	F	56	Cancer	Adenocarcinoma	T.S. Ltd. (Glasgow)
BH362	F	57	Cancer	Adenocarcinoma	B.C.C. (Glasgow)
125270584106	M	57	Cancer	Adenocarcinoma	S.H. (Swansea)
BH373	F	58	Cancer	Adenocarcinoma	B.C.C. (Glasgow)
SH179	F	58	Symptom. Control	N/A	S.H. (Swansea)
SH133	F	59	Symptom. Control	N/A	S.H. (Swansea)
PC3	F	60	Cancer	Adenocarcinoma	T.S. Ltd. (Glasgow)
PS210318	M	60	Symptom. Control	N/A	S.H. (Swansea)
SH196	M	60	Symptom. Control	Sigmoid polyp	S.H. (Swansea)
SH328	F	61	Cancer	Adenocarcinoma	S.H. (Swansea)
SH114	F	61	Symptom. Control	N/A	S.H. (Swansea)

BH401	M	62	Cancer	Adenocarcinoma	B.C.C. (Glasgow)
PC17	F	62	Cancer	Adenocarcinoma	T.S. Ltd. (Glasgow)
SH304	F	63	Cancer	Adenocarcinoma	S.H. (Swansea)
PC32	F	64	Cancer	Adenocarcinoma	T.S. Ltd. (Glasgow)
BJA080218	F	64	Symptom. Control	N/A	S.H. (Swansea)
ER270318	F	64	Symptom. Control	Colorectal polyp	S.H. (Swansea)
SH085	F	65	Cancer	Adenocarcinoma	S.H. (Swansea)
HS140218	M	65	Symptom. Control	N/A	S.H. (Swansea)
SM221018	M	65	Symptom. Control	CRC precursor	S.H. (Swansea)
SH153	F	66	Symptom. Control	N/A	S.H. (Swansea)
BH372	M	67	Cancer	Adenocarcinoma	B.C.C. (Glasgow)
1252672929	M	67	Cancer	Adenocarcinoma	S.H. (Swansea)
SH071	F	67	Symptom. Control	IBS	S.H. (Swansea)
SH172	F	67	Symptom. Control	N/A	S.H. (Swansea)
SH180	M	67	Symptom. Control	N/A	S.H. (Swansea)

Table S2 (cont.)

**ID**	**Sex**	**Age**	**Class**	**Diagnosis**	**Origin**
BH350	F	68	Cancer	Adenocarcinoma	B.C.C. (Glasgow)
SH139	M	68	Symptom. Control	N/A	S.H. (Swansea)
BH403	M	69	Cancer	Met Adenocarcinoma	B.C.C. (Glasgow)
125315237106	M	70	Cancer	Invasive IPMN	S.H. (Swansea)
SH077	M	70	Cancer	Adenocarcinoma	S.H. (Swansea)
SH214	M	70	Symptom. Control	Abdominal wall inflam.	S.H. (Swansea)
WEP071217	M	70	Symptom. Control	Familial adeno polyposis	S.H. (Swansea)
OD211217	M	71	Cancer	Met Adenocarcinoma	S.H. (Swansea)
1252642473	M	71	Symptom. Control	Rheumatoid lung nodule	S.H. (Swansea)
RM140418	M	71	Symptom. Control	Colorectal polyp	S.H. (Swansea)
SH185	F	72	Symptom. Control	N/A	S.H. (Swansea)
SH208	F	73	Symptom. Control	Chronic colitis	S.H. (Swansea)
SH276	F	74	Cancer	Adenocarcinoma	S.H. (Swansea)
SH320	F	75	Cancer	Met Adenocarcinoma	S.H. (Swansea)
SH330	M	75	Cancer	Adenocarcinoma	S.H. (Swansea)
JJ130218	F	75	Symptom. Control	Colorectal polyp	S.H. (Swansea)
SH178	M	75	Symptom. Control	Anaemia	S.H. (Swansea)
SH184	M	75	Symptom. Control	N/A	S.H. (Swansea)
BH361	M	76	Cancer	Met Adenocarcinoma	B.C.C. (Glasgow)
1252650177	F	77	Symptom. Control	Pulmonary embolism	S.H. (Swansea)
SH055	M	77	Symptom. Control	Crohn's disease	S.H. (Swansea)
SH113	F	78	Symptom. Control	IBS	S.H. (Swansea)
SH158	M	80	Cancer	Adenocarcinoma	S.H. (Swansea)
1252419390	F	81	Symptom. Control	Duodenal polyp	S.H. (Swansea)
G004	M	87	Cancer	Adenocarcinoma	G.H. (Gloucester)

Table S3. Cohort C; patients' information. Sorted by Age column. (M, males; F, females; T.S., Tissue Solutions; B.C.C., Beatson Cancer Centre; S.H., Singleton Hospital; G.H., Gloucestershire Hospital).

ID	Sex	Age	Class	Diagnosis	Origin
1252408663	M	28	Symptom. Control	N/A	S.H. (Swansea)
DOJ101117	M	36	Symptom. Control	Ulcerative colitis	S.H. (Swansea)
BH363	F	40	Cancer	Adenocarcinoma	B.C.C. (Glasgow)
KS120118	F	42	Symptom. Control	Colorectal polyp	S.H. (Swansea)
SH302	F	42	Symptom. Control	IBS	S.H. (Swansea)
TJ090218	M	42	Symptom. Control	Colorectal polyp	S.H. (Swansea)
PC9	F	45	Cancer	Adenocarcinoma	T.S. Ltd. (Glasgow)
PC10	F	48	Cancer	Adenocarcinoma	T.S. Ltd. (Glasgow)
PC24	F	48	Cancer	Adenocarcinoma	T.S. Ltd. (Glasgow)
PC1	M	50	Cancer	Adenocarcinoma	T.S. Ltd. (Glasgow)
PC19	M	50	Cancer	Adenocarcinoma	T.S. Ltd. (Glasgow)
PC23	F	50	Cancer	Adenocarcinoma	T.S. Ltd. (Glasgow)
BH354	F	51	Cancer	Met Adenocarcinoma	B.C.C. (Glasgow)
SH086	M	51	Cancer	Met Adenocarcinoma	S.H. (Swansea)
SH149	M	51	Symptom. Control	N/A	S.H. (Swansea)
PC11	M	51	Cancer	Adenocarcinoma	T.S. Ltd. (Glasgow)
PC22	F	51	Cancer	Adenocarcinoma	T.S. Ltd. (Glasgow)
PC29	F	51	Cancer	Adenocarcinoma	T.S. Ltd. (Glasgow)
SH144	M	52	Symptom. Control	N/A	S.H. (Swansea)
125254691606	M	53	Cancer	Met Adenocarcinoma	S.H. (Swansea)
125262256606	M	53	Cancer	Adenocarcinoma	S.H. (Swansea)
SH079	M	53	Cancer	Adenocarcinoma	S.H. (Swansea)
PC15	M	53	Cancer	Adenocarcinoma	T.S. Ltd. (Glasgow)
PC7	F	53	Cancer	Adenocarcinoma	T.S. Ltd. (Glasgow)
PC12	F	54	Cancer	Adenocarcinoma	T.S. Ltd. (Glasgow)
PC2	M	54	Cancer	Adenocarcinoma	T.S. Ltd. (Glasgow)
PC30	M	54	Cancer	Adenocarcinoma	T.S. Ltd. (Glasgow)
P128032	M	55	Cancer	Adenocarcinoma	T.S. Ltd. (Glasgow)
BH368	M	55	Cancer	Adenocarcinoma	B.C.C. (Glasgow)
SL260118	M	55	Symptom. Control	Polyp surveillance	S.H. (Swansea)
PC31	M	55	Cancer	Adenocarcinoma	T.S. Ltd. (Glasgow)
PC6	M	55	Cancer	Adenocarcinoma	T.S. Ltd. (Glasgow)
P128011	M	55	Cancer	Adenocarcinoma	T.S. Ltd. (Glasgow)
125310511306	M	56	Cancer	Adenocarcinoma	S.H. (Swansea)
SH078	M	56	Cancer	Adenocarcinoma	S.H. (Swansea)
PC16	F	56	Cancer	Adenocarcinoma	T.S. Ltd. (Glasgow)
PC25	M	56	Cancer	Adenocarcinoma	T.S. Ltd. (Glasgow)
BH362	F	57	Cancer	Adenocarcinoma	B.C.C. (Glasgow)
BH367	F	57	Cancer	Adenocarcinoma	B.C.C. (Glasgow)
125270584106	M	57	Cancer	Adenocarcinoma	S.H. (Swansea)
SH261	M	57	Cancer	NET	S.H. (Swansea)
TJ230118	F	57	Cancer	Met (Peritoneal) Carcinoma	S.H. (Swansea)
BH373	F	58	Cancer	Adenocarcinoma	B.C.C. (Glasgow)
SH179	F	58	Symptom. Control	N/A	S.H. (Swansea)
PC8	M	58	Cancer	Adenocarcinoma	T.S. Ltd. (Glasgow)

Table S3 (cont.)

ID	Sex	Age	Class	Diagnosis	Origin
SH133	F	59	Symptom. Control	N/A	S.H. (Swansea)
PC26	M	59	Cancer	Adenocarcinoma	T.S. Ltd. (Glasgow)
PS210318	M	60	Symptom. Control	N/A	S.H. (Swansea)
SH196	M	60	Symptom. Control	Sigmoid polyp	S.H. (Swansea)
PC13	M	60	Cancer	Adenocarcinoma	T.S. Ltd. (Glasgow)
PC28	M	60	Cancer	Adenocarcinoma	T.S. Ltd. (Glasgow)
PC3	F	60	Cancer	Adenocarcinoma	T.S. Ltd. (Glasgow)
P128002	F	60	Cancer	Adenocarcinoma	T.S. Ltd. (Glasgow)
P128010	F	61	Cancer	Adenocarcinoma	T.S. Ltd. (Glasgow)
SH328	F	61	Cancer	Adenocarcinoma	S.H. (Swansea)
SH114	F	61	Symptom. Control	N/A	S.H. (Swansea)
PC4	M	61	Cancer	Adenocarcinoma	T.S. Ltd. (Glasgow)
BH401	M	62	Cancer	Adenocarcinoma	B.C.C. (Glasgow)
PC14	M	62	Cancer	Adenocarcinoma	T.S. Ltd. (Glasgow)
PC17	F	62	Cancer	Adenocarcinoma	T.S. Ltd. (Glasgow)
1252886441	F	63	Cancer	Met Adenocarcinoma	S.H. (Swansea)
SH304	F	63	Cancer	Adenocarcinoma	S.H. (Swansea)
BH416	M	64	Cancer	Met Adenocarcinoma	B.C.C. (Glasgow)
BH445	F	64	Cancer	Adenocarcinoma	B.C.C. (Glasgow)
BJA080218	F	64	Symptom. Control	N/A	S.H. (Swansea)
ER270318	F	64	Symptom. Control	Colorectal polyp	S.H. (Swansea)
PC27	F	64	Cancer	Adenocarcinoma	T.S. Ltd. (Glasgow)
PC32	F	64	Cancer	Adenocarcinoma	T.S. Ltd. (Glasgow)
PC5	M	64	Cancer	Adenocarcinoma	T.S. Ltd. (Glasgow)
BH418	F	65	Cancer	Adenocarcinoma	B.C.C. (Glasgow)
SH085	F	65	Cancer	Adenocarcinoma	S.H. (Swansea)
HS140218	M	65	Symptom. Control	N/A	S.H. (Swansea)
SM221018	M	65	Symptom. Control	CRC precursor	S.H. (Swansea)
PC20	F	65	Cancer	Adenocarcinoma	T.S. Ltd. (Glasgow)
BH353	F	66	Cancer	Adenocarcinoma	B.C.C. (Glasgow)
SH309	F	66	Cancer	NET	S.H. (Swansea)
SH153	F	66	Symptom. Control	N/A	S.H. (Swansea)
PC18	M	66	Cancer	Adenocarcinoma	T.S. Ltd. (Glasgow)
PC21	M	66	Cancer	Adenocarcinoma	T.S. Ltd. (Glasgow)
BH372	M	67	Cancer	Adenocarcinoma	B.C.C. (Glasgow)
BH422	M	67	Cancer	Adenocarcinoma	B.C.C. (Glasgow)
BH448	M	67	Cancer	Adenocarcinoma	B.C.C. (Glasgow)
1252672929	M	67	Cancer	Adenocarcinoma	S.H. (Swansea)
SH071	F	67	Symptom. Control	IBS	S.H. (Swansea)
SH172	F	67	Symptom. Control	N/A	S.H. (Swansea)
SH180	M	67	Symptom. Control	N/A	S.H. (Swansea)
BH350	F	68	Cancer	Adenocarcinoma	B.C.C. (Glasgow)
125289145806	M	68	Cancer	NET	S.H. (Swansea)
SH139	M	68	Symptom. Control	N/A	S.H. (Swansea)
BH403	M	69	Cancer	Met Adenocarcinoma	B.C.C. (Glasgow)
1252640380	M	69	Cancer	Liposarcoma	S.H. (Swansea)
1252899776	F	69	Cancer	Adenocarcinoma	S.H. (Swansea)

Table S3 (cont.)

ID	Sex	Age	Class	Diagnosis	Origin
125315237106	M	70	Cancer	Invasive IPMN	S.H. (Swansea)
SH077	M	70	Cancer	Adenocarcinoma	S.H. (Swansea)
SH214	M	70	Symptom. Control	Abdominal wall inflam.	S.H. (Swansea)
WEP071217	M	70	Symptom. Control	Familial adeno polyposis	S.H. (Swansea)
BH413	F	71	Cancer	Met Adenocarcinoma	B.C.C. (Glasgow)
OD211217	M	71	Cancer	Met Adenocarcinoma	S.H. (Swansea)
1252642473	M	71	Symptom. Control	Rheumatoid lung nodule	S.H. (Swansea)
RM140418	M	71	Symptom. Control	Colorectal polyp	S.H. (Swansea)
P128017	M	71	Cancer	Adenocarcinoma	T.S. Ltd. (Glasgow)
BH406	M	72	Cancer	Met Adenocarcinoma	B.C.C. (Glasgow)
BH414	F	72	Cancer	Adenocarcinoma	B.C.C. (Glasgow)
SH185	F	72	Symptom. Control	N/A	S.H. (Swansea)
1252813381	M	73	Cancer	Adenocarcinoma	S.H. (Swansea)
SH225	M	73	Cancer	NET	S.H. (Swansea)
SH269	M	73	Cancer	NET	S.H. (Swansea)
SH314	M	73	Cancer	Invasive IPMN	S.H. (Swansea)
SH208	F	73	Symptom. Control	Chronic colitis	S.H. (Swansea)
1252409748	M	74	Cancer	Adenocarcinoma	S.H. (Swansea)
SH276	F	74	Cancer	Adenocarcinoma	S.H. (Swansea)
125312955206	M	75	Cancer	NET	S.H. (Swansea)
RT101117	M	75	Cancer	Adenocarcinoma	S.H. (Swansea)
SH313	M	75	Cancer	Adenocarcinoma	S.H. (Swansea)
SH320	F	75	Cancer	Met Adenocarcinoma	S.H. (Swansea)
SH322	M	75	Cancer	Met Adenocarcinoma	S.H. (Swansea)
SH330	M	75	Cancer	Adenocarcinoma	S.H. (Swansea)
JJ130218	F	75	Symptom. Control	Colorectal polyp	S.H. (Swansea)
SH178	M	75	Symptom. Control	Anaemia	S.H. (Swansea)
SH184	M	75	Symptom. Control	N/A	S.H. (Swansea)
BH361	M	76	Cancer	Met Adenocarcinoma	B.C.C. (Glasgow)
BH434	F	76	Cancer	Met Adenocarcinoma	B.C.C. (Glasgow)
CT091117	F	76	Cancer	Met Adenocarcinoma	S.H. (Swansea)
SH065	F	76	Cancer	Adenocarcinoma	S.H. (Swansea)
SH203	F	76	Cancer	Adenocarcinoma	S.H. (Swansea)
1252650177	F	77	Symptom. Control	Pulmonary embolism	S.H. (Swansea)
SH055	M	77	Symptom. Control	Crohn's disease	S.H. (Swansea)
SH113	F	78	Symptom. Control	IBS	S.H. (Swansea)
1252341008	M	80	Cancer	Adenocarcinoma	S.H. (Swansea)
1252637561	F	80	Cancer	Met Adenocarcinoma	S.H. (Swansea)
SH158	M	80	Cancer	Adenocarcinoma	S.H. (Swansea)
1252419390	F	81	Symptom. Control	Duodenal polyp	S.H. (Swansea)
1252812461	F	83	Cancer	Adenocarcinoma	S.H. (Swansea)
125234430306	M	83	Cancer	Met Adenocarcinoma	S.H. (Swansea)
G004	M	87	Cancer	Adenocarcinoma	G.H. (Gloucester)

Table S4. Summary table on age and sex information about all the classes used to perform analysis. (F, females; M, males).

Age	Cancer (F/M)	Healthy Control (F/M)	Symptomatic Control (F/M)
20+	0 / 0	0 / 0	0 / 1
30+	0 / 0	0 / 1	0 / 1
40+	4 / 0	1 / 0	2 / 1
50+	11 / 22	6 / 20	2 / 3
60+	17 / 16	15 / 20	6 / 6
70+	8 / 16	19 / 15	5 / 7
80+	2 / 4	2 / 1	1 / 0
**Total**	100	100	35

Table S5. Top 15 wavenumbers from random forest (RF) classification of Cohort A with tentative assignments (where ν_s = symmetrical stretching, ν_{as} = asymmetrical stretching and δ = bending).

Wavenumber (cm^{-1})	Σ Gini	Tentative Assignment
1555.5	100.0	Amide II of proteins δ(NH)/ν(CN)
1563.5	55.2	
1547.5	41.7	
1515.5	21.6	
1507.5	18.2	
1619.5	15.1	Amide I of proteins ν(CO)/ν(CN)/δ(NH)
1571.5	13.3	Amide II of proteins δ(NH)/ν(CN)
1707.5	12.6	ν_s(CO) of lipids
1523.5	9.8	Amide II of proteins δ(NH)/ν(CN)
1499.5	8.3	
1195.5	6.0	ν_{as}(CO-O-C) of carbohydrates
1627.5	5.9	Amide I of proteins ν(CO)/ν(CN)/δ(NH)
1155.5	5.9	ν_{as}(CO-O-C) of carbohydrates
1691.5	5.4	Amide I of proteins ν(CO)/ν(CN)/δ(NH)
1715.5	5.3	ν_s(CO) of lipids

Table S6. Top 15 wavenumbers from random forest (RF) classification of Cohort B with tentative assignments (where ν_s = symmetrical stretching, ν_{as} = asymmetrical stretching and δ = bending).

Wavenumber (cm^{-1})	Σ Gini	Tentative Assignment
1499.5	100.0	Amide II of proteins δ(NH)/ν(CN)
1267.5	69.4	$\nu_{as}(PO_2^-)$ of nucleic acids
1491.5	64.7	Amide II of proteins δ(NH)/ν(CN)
1507.5	44.8	
1483.5	34.5	
1259.5	33.5	$\nu_{as}(PO_2^-)$ of nucleic acids
1555.5	28.9	Amide II of proteins δ(NH)/ν(CN)
1027.5	17.8	ν_s(CO-O-C) of carbohydrates
1515.5	25.6	Amide II of proteins δ(NH)/ν(CN)
1523.5	24.1	
1051.5	21.7	ν_s(CO-O-C) of carbohydrates
1275.5	21.3	$\nu_{as}(PO_2^-)$ of nucleic acids
1251.5	20.8	
1035.5	19.5	ν_s(CO-O-C) of carbohydrates
1107.5	17.5	$\nu_s(PO_2^-)$ of nucleic acids

Table S7. Statistical performances of random forest (RF), partial least squares - discriminant analysis (PLS-DA), support vector machine (SVM) and receiver operating characteristic (ROC) analysis per-formed on Cohort C. (AUC, area under the ROC curve; SD, standard deviation).

Cohort C	RF			PLS-DA			SVM		
Sensitivity ± SD (%)	87.4	±	6.4	72.7	±	9.7	79.1	±	6.9
Specificity ± SD (%)	51.4	±	13.1	87.3	±	11.0	77.3	±	15.5
Accuracy ± SD (%)	69.4	±	6.3	80.0	±	7.0	78.2	±	7.5
**ROC** (AUC)	0.835			0.849			0.845		

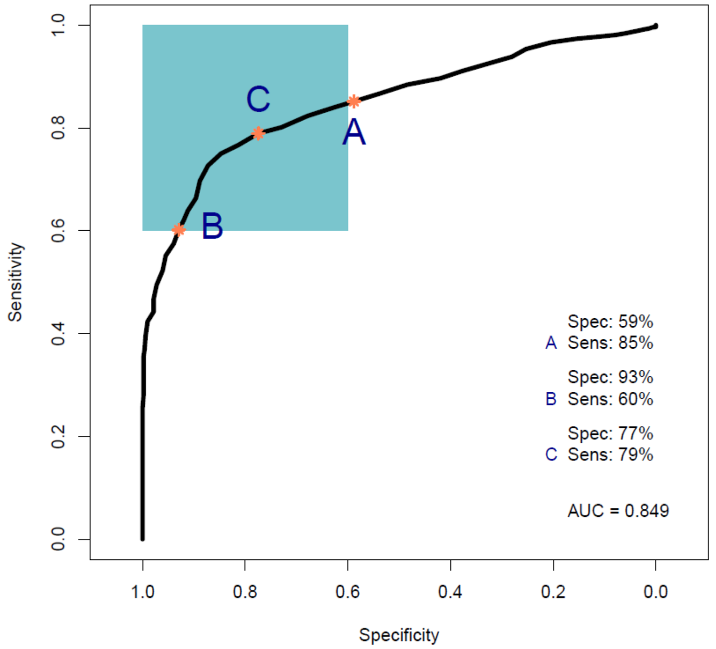

Figure S1. Receiver operating characteristic (ROC) curve analysis calculated from a partial least squares - discriminant analysis (PLS-DA) model performed on Cohort C. The colored box indicates the region with sensitivity and specificity greater than 60%. The orange dots indicate the points

within that region that have maximum sensitivity (A), maximum specificity (B), and balanced sensitivity and specificity (C). (Sens, sensitivity; Spec, specificity; AUC, area under the ROC curve).

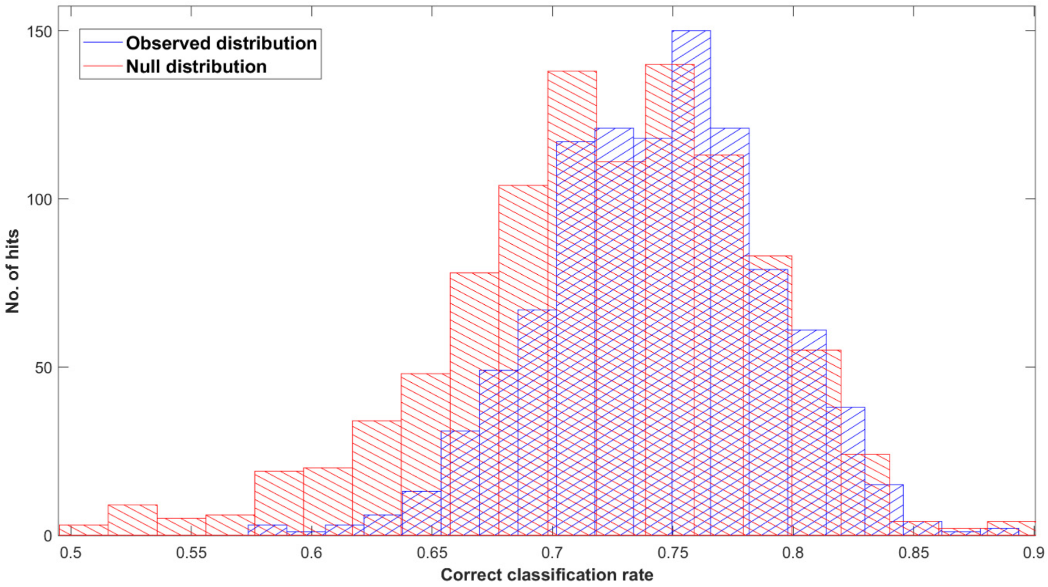

Figure S2. Permutation tests' output plot; null (red) and observed (blue) distribution classification rated for Cohort C with a partial least squares - discriminant analysis (PLS-DA) classification model after 1,000 bootstraps, using pre-processed spectra.
